# Risk Behaviours Associated with Dating and Relationship Violence among 11–16 Year Olds in Wales: Results from the 2019 Student Health and Wellbeing Survey

**DOI:** 10.3390/ijerph18031192

**Published:** 2021-01-29

**Authors:** Danielle V. R. Couturiaux, Honor Young, Rebecca E. Anthony, Nicholas Page, Emily Lowthian, G. J. Melendez-Torres, Gillian Hewitt, Graham F. Moore

**Affiliations:** 1 Centre for Development, Evaluation, Complexity and Implementation in Public Health Improvement (DECIPHer), School of Social Sciences, Cardiff University, 1–3 Museum Place, Cardiff CF10 3BD, UK; YoungH6@cardiff.ac.uk (H.Y.); PageN2@cardiff.ac.uk (N.P.); LowthianEM@cardiff.ac.uk (E.L.); HewittG@cardiff.ac.uk (G.H.); MooreG@cardiff.ac.uk (G.F.M.); 2 College of Medicine and Health, South Cloisters, St Luke’s Campus, University of Exeter, Heavitree Road, Exeter EX1 2LU, UK; g.j.melendez-torres@exeter.ac.uk

**Keywords:** young people, dating violence, substance use, sexting, bullying, cyberbullying

## Abstract

(1) Background: This study examines the associations between risk behaviours and adolescent emotional and physical dating and relationship violence (DRV) victimisation and perpetration, and how these vary by gender. The risk behaviours explored include bullying, cyberbullying, sexting, alcohol, and cannabis use; (2) Methods: Cross-sectional self-report data from the School Health Research Network (SHRN) 2019 Student Health Wellbeing (SHW) survey of 48,397 students aged 11–16 from 149 schools across Wales were analysed using single and multiple-behaviour logistic regression models to explore the associations between each risk behaviour and emotional and physical DRV victimisation and perpetration; (3) Results: Bivariate analyses revealed a statistically significant association between DRV and all risk behaviours. In multivariate analyses, students who reported bullying, cyberbullying, sexting, and substance use, compared to those that had not, had significantly higher odds of experiencing and perpetrating emotional and physical DRV; and (4) Conclusions: Future studies on DRV should consider a mixed-methods approach to explore the context in which DRV and risk behaviours interrelate. Results from this study indicate the possibility that prevention and intervention programmes in school settings that seek to develop healthy school environments and peer-to-peer relationships, could inadvertently reduce the occurrence of future DRV and associated risk behaviours.

## 1. Introduction

The term domestic violence encompasses emotional, financial, sexual, psychological, and/or physical abuse [1]. While the legal definitions of domestic violence in the UK do not apply to adolescents under the age of 16 [2], a growing body of research demonstrates that adolescent dating and relationship violence (DRV) is occurring not only with much higher prevalence than previously expected, but also to younger people, with some studies reporting similar rates for both girls and boys [3,4,5,6,7,8,9,10,11].

Drawing on a large European study of 14 to 17 year olds, 48% of girls and 27% of boys in England reported having experienced emotional DRV and 22% of girls and 12% of boys had experienced physical DRV [7]. Research using a similar age group in England, Scotland, and Wales, found that 11% of girls and 4% of boys had been victims of severe physical DRV [4], while a recent study in England and Wales, with a slightly older age group, found that over half of the 16 to 19 year olds surveyed had experienced some form of DRV in their lifetime [8]. In one of the few UK (and international) studies to explore both emotional and/or physical DRV victimisation and/or perpetration, Young et al. [9] counter the preconceived notion that DRV is perpetrated mostly by boys against girls. Here the authors demonstrated that boys and girls in Wales were equally likely to perpetrate emotional (16% and 18% respectively) and physical DRV (7% and 8% respectively), while nearly a third of girls and a fifth of boys had experienced emotional DRV victimisation; although a slightly higher percentage of boys was found to have experienced physical DRV victimisation, compared to girls (17% vs. 12%).

Although domestic violence and DRV share similar characteristics, such as aspects of emotional, physical and sexual abuse, the word ‘domestic’ in domestic violence tends to associate the abuse as occurring within household settings, not just between romantic partners but also household members (e.g., parent and child). Because young people under the age of 16 are unlikely to be cohabitating, DRV, unlike domestic violence, focuses more on violence which occurs within romantic relationships and as such, DRV is more akin to, or used interchangeably with, intimate partner violence and abuse (IPVA). Additionally, although the age limit defining domestic abuse in the UK hinders the acknowledgement and recognition of DRV among those under the age of 16, the United Nations Convention on the Rights of the Child [12] mandates that it is the duty of States to protect children from harm. In response, the Welsh Government developed a number of legislative measures, including the Rights of Children and Young Persons (Wales) Measure [13], the Violence Against Women, Domestic Abuse and Sexual Violence (Wales) Act (VAWD ASV) [14] and more recently the delivery of comprehensive and inclusive Relationships and Sexuality Education (RSE), including a focus on healthy relationships [15,16].

The Home Office estimates the social and economic cost of domestic violence in England and Wales at around £66 billion [17]; and this would perhaps be higher if it included impacts of DRV within adolescent relationships. Adolescent DRV has been identified as a precursor for adult domestic violence, with youth who experience DRV victimisation significantly more likely to report domestic violence in adulthood [18,19,20]. Likewise, a recent UK cohort study found 8% of respondents reported IPVA victimisation and 3–4% IPVA perpetration, before and after the age of 18 respectively [11]. Additionally, a systematic review exploring the temporal impacts of physical, sexual, and psychological DRV victimisation and/or perpetration, also reported links between DRV and future revictimisation and perpetration, as well as poor mental health outcomes (e.g., depression and suicidality); negative physical health, including risky sexual behaviours (e.g., unprotected sex); and substance misuse [21]. There are also important gender variations in the impacts of experiencing DRV [3,5]; for example, girls experience a more negative reaction (e.g., feeling scared or upset) compared to boys [6,7,11,22,23,24], while boys are more likely than girls to report a positive only impact (e.g., found it funny) or no impact [6,7,11,24]. The adverse psychological, social, and economic consequences associated with DRV provide rationale for prevention and intervention. Researchers have attempted to identify risk factors predictive of adolescent DRV to help inform adolescent DRV intervention and prevention programmes.

### 1.1. Alcohol

Alcohol has been consistently associated with adolescent DRV [25,26,27,28,29,30,31]. Significant associations have been found internationally across a wide range of cross-sectional, longitudinal and nationally representative studies, despite variations in the measurement of alcohol consumption (e.g., binge drinking, heavy alcohol consumption, recent alcohol use, and age of first alcohol use) and regardless of DRV victimisation or perpetration. Previous qualitative [32,33,34], experimental [35], and cross-sectional [26,36] research studies have argued that the properties of alcohol, depending on the person, can both weaken cognitive functions and disorient the user, which can make a person more vulnerable to victimisation, and exacerbate aggression, thus making the substance user more likely to perpetrate aggression. Moreover, quantitative research on college students has also found evidence to suggest alcohol intoxication can increase the risk of perpetrating IPVA [37,38], findings which have also been found in qualitative studies [32,33,34] on substance use and DRV among adolescents and young people.

### 1.2. Cannabis

Evidence on the association between cannabis and DRV is equivocal. US research exploring the proximal and time-varying effects of cannabis on physical DRV perpetration cross-sectionally found an association between cannabis consumption and physical DRV perpetration for girls only, however longitudinally, this association was no longer significant [28]. Likewise, other longitudinal US research also found that cannabis use at baseline (age 15) was not predictive of physical DRV perpetration a year later [30]. In a qualitative study of adolescents aged 14–19 from community-based organisations in Hawaii, Baker [33] found evidence to suggest the calming effects of cannabis acted as a deterrent to DRV, while unlike alcohol, cannabis was not used to deal with the negative effects of DRV. On the other hand, a UK longitudinal study found that males and females who reported regular cannabis use at age 16 were around twice as likely as those not using cannabis to report experiencing or perpetrating IPVA by age 21 [11]. Other studies have found that adolescents who report any form of DRV, compared to those who have not, had a higher mean score for frequency of cannabis use [27]. Similarly, physical and verbal DRV victimisation have also been significantly associated with cannabis consumption [26].

### 1.3. Bullying

Existing evidence suggests a pattern between bullying perpetration and DRV. A cross-sectional study in Canada with students aged 9–15 years, found that bullies, compared to non-bullies, started dating at an earlier age, reported lower mean scores for relationship intimacy, commitment, and affection, and were more likely to display aggressive behaviours towards their partners as well as experience DRV victimisation [39]. Similarly, longitudinal US research found that 16% of sixth graders who directly bullied others had perpetrated physical DRV by the eighth grade, compared to 8% of students who had not directly bullied another person [40]. Although no evidence was found to suggest these associations varied by gender, Canadian research with 9th–11th graders found that bullying perpetration was significantly associated with DRV perpetration for boys only [41]. However, the latter study did not distinguish by type of DRV. 

Bullying victimisation on the other hand, has been associated with increased odds for emotional and sexual DRV victimisation but not physical or online DRV victimisation [24]. Similarly, Debnam et al. [42] in their cross-sectional US study found that adolescents who had been bullied had higher odds of reporting emotional and physical DRV victimisation. Despite these associations, research in this area is limited, particularly in the UK. More research is required that explores the relationship between bullying victimisation and perpetration and different forms of DRV using the same sample.

### 1.4. Cyberbullying

The way adolescent DRV is understood is continuously being redefined. More recently, the Centre for Disease Control and Prevention [43] definition acknowledged that DRV can occur face-to-face or remotely using technological devices, the latter being referred to as cyber dating violence [44,45] or technology assisted adolescent dating violence and abuse [46]. Much like violence within adolescent relationships is comparable to bullying among peers, so is cyber dating violence and cyberbullying [44]. Although research in this area is scarce, associations have been found between experiencing cyber dating violence and cyberbullying victimisation [45]. 

The few studies that have explored the relationship between cyberbullying and face-to-face DRV, have found significant associations between cyberbullying victimisation and sexual DRV victimisation [47]. Moreover, cross-sectional research with 7th–12th grade US students identified a moderate association between emotional and physical DRV perpetration and cyberbullying perpetration, and physical DRV victimisation and cyberbullying victimisation, in addition to a moderately strong relationship between emotional DRV victimisation and cyberbullying victimisation [48]. The lack of research on cyberbullying and DRV in the UK warrants further exploration on this topic. 

### 1.5. Sexting

The majority of studies on adolescents and sexting have defined sexting as the use of digital technologies to send and/or receive sexual texts, images or videos online [49]. Research across five European countries with adolescents aged 14–17, found that nearly two fifths of English students reported sexting; the highest proportion across the five countries [50]. Participants who had experienced some form of DRV were more likely than non-victims to have shared a sexually explicit image with their partner. Similar findings between DRV victimisation and sexting were found among young people in England and Wales, with males and females who had reported sexting between 2 to 8 and 2 to 4 times more likely to have experienced DRV compared to those that had not sexted [8]. In England, there are large gender disparities between girls (42%) and boys (13%) reporting having an image shared by a partner without prior consent, with girls more likely than boys to report negative feelings following the incident [50]. Additionally, over a quarter of teenage girls surveyed in England reported sending a sexually explicit image because they felt coerced into doing so.

The high prevalence of sexting among teenagers who experience DRV is of concern as they are at risk of blackmail or humiliation by having images publicly shared without their permission (e.g., revenge porn) [8,46,50,51,52,53,54,55,56]. While the majority of teenagers report positive experiences from sexting in their romantic relationships [50], there are implications relating to the creation, distribution or receipt of photographs due the legal definition of child pornography (i.e., indecent images of someone under the age of 18), as well as the sharing of images without the consent of the sender [53,55,57]. This is not only due to the negative repercussions for the victim but also the danger of the pictures shared online being repurposed as child pornography [53,55,57]. Such is the association between sexting and DRV, that having a sexually explicit image shared by a partner without prior permission has been classed as a type of cyber dating violence, a form of electronic sexual DRV [43,44].

### 1.6. Current Study

Given the paucity of research in the UK on DRV using nationally representative samples, this paper aims to expand on research by Young et al. [9] by identifying risk factors associated with adolescent DRV. Through an analysis of the current UK and international literature, the following risk behaviours have been identified for analysis: alcohol use, cannabis use, bullying, cyberbullying, and sexting. Studies which have explored these risk factors individually can only account for a small portion of a complex problem [3]. As adolescence is a period in which teens often experiment and take part in adult-like behaviours it is likely that numerous risk behaviours, including different types of DRV, will cluster together [3,5,58]. Thus, this study explores associations between four types of adolescent DRV as well as risk-taking behaviours that could be connected to DRV, in order to identify any possible groups or characteristics that may indicate a greater vulnerability to perpetrating or being a victim of DRV. Given some of the variations in findings by gender in the current DRV literature, data will be analysed separately by gender. The research will explore the following research questions:

RQ1—How do risk behaviours associate with different types of DRV? 

RQ2—Do associations between risk behaviours and types of DRV vary by gender and type of violence?

## 2. Methods

This study uses cross-sectional data obtained from 11 to 16 year olds in Wales, UK via the School Health Research Network (SHRN) 2019 Student Health and Wellbeing (SHW) survey, which includes questions from the 2017/18 Health Behaviour in School-aged Children (HBSC) survey. Data were collected using an online, self-completion questionnaire divided into four survey routes, with questions randomly allocated to appear within singular or multiple routes to ensure nationally representative data across a wide range of diverse topics. A total of 119,388 students from 198 schools, including 94% of all maintained secondary schools, completed the 2019 SHW survey. Questions on dating and relationships were asked to around three-quarters of the sample (*n* = 91,084, 149 schools). Further information on survey methodology can be found at www.shrn.org.uk/national-data/.

For the purposes of this study only those students who had ever dated someone and answered the relevant questions are included in the analysis. Although one of the aims of this study was to explore how risk behaviours associated with DRV varied by gender, students who answered ‘are you a boy or a girl?’ with ‘neither word describes me’ are excluded as the sample size for this measure is too small for the analyses carried out in this study. Additionally, students who selected ‘I do not want to answer’, an option for all questions in the survey, including gender, are also excluded from the analysis. Thus, the initial dataset of 80,573 boys and girls, was reduced to the 48,397 who had ever dated someone.

### 2.1. Measures

#### 2.1.1. Socio-Demographic Characteristics 

Socioeconomic Status—The Family Affluence Scale (FAS) [59] was used to measure family socioeconomic status (SES) with scores divided into equal tertiles and categorized as low, medium, and high SES.

Ethnicity—Students could select ‘White British’, ‘White Irish’, ‘White-Gypsy/Traveller’, ‘Mixed or Multiple Ethnic Group’, ‘Pakistani’, ‘Indian’, ‘Bangladeshi’, ‘Chinese’, ‘African’, ‘Caribbean or Black’, ‘Arab’, and ‘Other’. To enable meaningful analysis of ethnic trends, responses were collapsed into ‘White’ and ‘Black, Asian and minority ethnic people’ to address smaller sample sizes for some ethnicities. 

Household Composition—Participants reported who they live with: Mother; Father; Mother’s partner; Father’s partner; Grandparent(s); Aunt(s)/Uncle(s); Adult brother(s) and/or sister(s); Foster parents; I live in residential care or a children’s home; I live independently (on my own or with friends or my partner); Someone or somewhere else. Variables were collapsed into both parents, single parent, stepfamily, and other. 

#### 2.1.2. Dating and Relationship Violence (DRV) 

Dating Experience—Only students who had responded ‘yes, with a girl(s)’ and/or ‘yes, with a boy(s)’ to ‘have you ever been ‘seeing’ someone, ‘dating’ or ‘going out with’ someone?’, were asked questions on DRV. 

Emotional DRV—Students were asked whether a partner had ever made ‘hurtful comments’ towards them (victimisation) and whether they had ever made ‘hurtful comments’ towards a partner (perpetration). 

Physical DRV—Measures for physical DRV in the SHW survey distinguished between ‘pushed, shoved, or slapped’ and ‘punched, kicked, or beat-up’. For each, students were asked whether they had ever experienced and/or perpetrated physical DRV in their romantic relationships. Due to low response rates, answers for ’pushed, shoved, or slapped’ and ‘punched, kicked, or beat-up’ were combined [9]. 

Response options for the SHW DRV measures included ‘never’, ‘once, ‘a few times’, and ‘often’. Responses were collapsed to provide binary ‘yes’ and ‘no’ indicators for emotional and physical DRV victimisation and perpetration. 

#### 2.1.3. Risk Behaviour Measures 

Bullying Victimisation and Perpetration—Students were asked ‘how often have you been bullied at school’ (bullying victimisation) and ‘how often have you taken part in bullying another person(s)’ (bullying perpetration) in the past two months. 

Cyberbullying Victimisation—Participants were asked if in the last two months had they ever been cyberbullied ‘e.g., someone sent mean instant messages, email or text messages about you, wall postings, created a website making fun of you, posted unflattering or inappropriate pictures of you online without permission or shared them with others’. 

Response options for bullying and cyberbullying included ‘I have not bullied another person/been bullied/been cyberbullied’, ‘it has happened once or twice’, ‘2 or 3 times a month’, ‘about once a week’, and ‘several times a week’. Answers were dichotomised into ‘yes’ and ‘no’ answers. 

Sexting—Students were asked if they had ever ‘sent someone a sexually explicit image of yourself’ and if ‘anyone ever sent, forwarded or shared a sexually explicit image of you to other people, without asking’. Participants were asked the second question regardless of whether they had ever sexted. ‘Once’ or ‘more than once’ responses were collapsed to form binary (‘yes’, ‘no’) measures of ever sexted and ever had an image shared without permission.

Alcohol—Students were asked on the days when they did drink alcohol, how many drinks did they usual have. Responses for this question were coded as ‘<1 drink’, which includes those that reported never drinking alcohol, ‘1–4 drinks’, and ‘≥5 drinks’.

Cannabis—Responses options for ‘at what age did you first use cannabis?’ ranged between 11 years old or less and 18 years old or older, as well as a ‘never’ option. Responses for the ages of 11 years old or less to 16 were collapsed and coded as ‘yes’ to ever having tried cannabis, whereas ‘never’ was coded as ‘no’. Any participants who selected 17 or 18 years old or older were excluded due to the low likelihood that someone in the oldest year group, year 11, would be this old.

### 2.2. Data Analyses

All statistical analyses were carried out using STATA v.14 [60]. Student socio-demographic characteristics were first explored descriptively, and single-behaviour logistic regression models used to assess whether associations between individual risk behaviours and each DRV-related dependent variable were statistically significant by gender and overall. All multi-behaviour models included school grade, SES, family structure and ethnicity as covariates. Subgroup analyses were undertaken by gender and overall. As mixed-effects or multilevel models, which generate ‘within-school’ or cluster-specific estimates, or models with school-clustered standard errors, would underestimate the potential impact of school contexts, analyses include population-average estimates. As such, to provide a population-average coefficient while accounting for school-level clustering, all regression models were undertaken using generalized estimating equations (GEE), logit link, exchangeable correlation matrices, and robust standard errors [9]. 

### 2.3. Research Ethics and Consent

Ethical approval for this study was granted by Cardiff University School of Social Sciences Research Ethics Committee. Informed consent was obtained from schools, parents, and students. Schools had to register to take part in the survey, parents had the option to withdraw their child(ren) from data collection, while students’ participation was optional, with the first question in the survey asking for their consent to take part.

## 3. Results

Of the 48,397 students that reported ever dating someone, 50.6% were girls. As seen in Table 1, the majority of respondents reported their ethnicity as White (92.2%) and living with both parents (59.5%).

### 3.1. Prevalence of Dating and Relationship Violence

Emotional DRV victimisation was the most common form reported, with 30.0% of girls and 23.6% of boys experiencing this form of DRV (Table 2). Roughly the same proportion of boys and girls reported perpetrating emotional DRV (18.1% and 19.3% respectively). A larger proportion of boys than girls reported experiencing physical DRV (19.3% and 12.8% respectively), whereas similar proportions of boys and girls, around 8.3%, reported perpetrating physical DRV.

### 3.2. Gender Differences

While girls were more likely than boys to report having experienced emotional victimisation (OR = 1.39, *p* < 0.001, 95% CI = [1.33–1.45]), they were less likely than boys to report physical DRV victimisation (OR = 0.62, *p* < 0.001, 95% CI = [0.58–0.65]). The odds for girls reporting perpetrating emotional DRV, compared to boys, were similar (OR = 1.08, *p* < 0.01, 95% CI = [1.02–1.14]). Results for gender associations for physical DRV perpetration were not statistically significant (OR = 1.04, *p* > 0.05, 95% CI = [0.97–1.11]).

### 3.3. Multivariate Analysis

Table 3 shows the adjusted odds ratios (and 95% confidence intervals) from the logistic regression models between bullying, substance use, and sexting and each type of DRV, while controlling for grade, SES, ethnicity, and family structure and stratifying by gender. Further details, including unadjusted odds ratios, and adjusted odds ratios for boys and girls combined, can be found in Online Supplement, Tables S1 and S2.

For boys and girls alike, having been bullied, bullying another person, experiencing cyberbullying, sexting, alcohol consumption, and ever having tried cannabis, significantly increased the odds of experiencing and perpetrating emotional and physical DRV even when controlling for other risk behaviours and socio-demographic characteristics.

.

#### 3.3.1. Emotional DRV Victimisation

Boys and girls who had experienced cyberbullying were around twice as likely to have reported experiencing emotional DRV, compared to those that had not. Compared to those that had not, boys and girls who had reported both ever having sexted and having a sexually explicit image shared without prior consent, were roughly three times more likely to have experienced emotional DRV.

#### 3.3.2. Physical DRV Victimisation

Like emotional DRV victimisation, boys who had reported cyberbullying and both ever having sexted and having a sexually explicit image shared without prior consent had significantly higher odds of having experienced physical DRV victimisation. Girls who reported sexting and having an image shared without permission, compared to those that had not, were over three times as likely to have experienced physical DRV, while girls who had reported drinking five alcoholic drinks or more in one sitting, compared to those that had less than one alcoholic drink, were twice as likely to have reported experiencing physical DRV victimisation.

#### 3.3.3. Emotional DRV Perpetration

The odds of perpetrating emotional DRV were around twice as high for boys and girls who had reported bullying another person compared to those that had not, and three times higher for those that had reported ever sexting and having a sexually explicit image shared without prior consent.

#### 3.3.4. Physical DRV Perpetration

Girls who reported drinking five or more drinks in one sitting had 2.37 times the odds of reporting perpetrating physical DRV compared to those that had not. The odds of perpetrating physical DRV were slightly higher for boys than girls who had reported bullying another person compared to those that had not. In contrast, the odds of reporting physical DRV perpetration were higher for girls than boys who had ever sexted and had a sexually explicit image shared without permission.

## 4. Discussion

Using multiple regression models, this paper was able to identify risk behaviours associated with DRV, as well as how these associations varied by type of DRV and gender. Our findings contribute to the existing research exploring DRV prevalence and associations between DRV and socio-demographic characteristics [9]. Comparing previous SHW survey responses from 2017 [9] with the current 2019 data, results indicated a slight increase in the prevalence rates for emotional and physical DRV perpetration and victimisation. Yet like previous UK research [7,8,9,10,11], the most common form of DRV was emotional victimisation. 

In line with previous studies [24,39,40,41,48,61], bullying victimisation and perpetration were significantly associated with DRV victimisation and perpetration, however the odds of reporting emotional and physical DRV perpetration, as opposed to victimisation, were higher among students who had bullied another person compared to those that had not. Likewise, students who reported being bullied compared to those that had not, had higher odds of experiencing DRV compared to perpetrating DRV. Leadbeater, Connolly and Temple [62] argue that the use of violence in peer relationships through bullying as an acceptable means of conflict resolution can evolve and translate into romantic relationships later in adolescence and young adulthood. The reasons why students who reported bullying victimisation likely also reported DRV victimisation are less clear. However, previous research on children and young people has demonstrated how victimisation in one context can link to repeat victimisation as well as victimisation across different contexts, particularly for those that experience multiple forms of victimisation [63]. This could help explain why, for example, women reporting IPVA were also likely to report peer victimisation and childhood sexual abuse [64].

Similar to previous studies on alcohol and DRV [25,26,27,29,30,31], students who reported higher episodic drinking, compared to those that did not drink alcohol, had increased odds of experiencing or perpetrating DRV. This trend was particularly evident among girls who drank five or more alcoholic drinks in one sitting when compared to girls who did not report drinking alcohol, as they were around twice as likely to report physical DRV victimisation and perpetration. Nevertheless, previous Canadian longitudinal research with 12 to 18 year olds demonstrated that although peer relational and physical abuse predicted DRV perpetration, binge drinking only mediated the link between early peer physical abuse and future DRV perpetration, and not early peer relational abuse [65]. Given the diversity of drinking cultures internationally [66], further longitudinal and qualitative studies are needed to explore the relationship between alcohol, DRV, and bullying among children and young people in the UK.

Like alcohol, the association between cannabis and DRV was stronger among girls than boys. However, given the equivocal nature of the existing literature in this topic area, further studies are warranted to qualitatively explore the context in which cannabis consumption occurs within abusive relationships; particularly explanations for the gender differences identified. Findings from previous longitudinal studies [67,68,69] suggest that girls who mature earlier are more likely to socialise with older peers, engage in higher risk behaviours, including cannabis use, and have dating experience, likely with older partners. The latter in turn may increase their risk of DRV as previous research reported that girls that date older men are at a higher risk of DRV [4].

Consistent with previous research [8,50,70], sexting was the strongest predictor of experiencing and perpetrating emotional and physical DRV. The odds of experiencing or perpetrating DRV were significantly higher for students who had ever reported sexting and had had a sexually explicit image shared without their consent, compared to those that had not. Research by Lippman and Campbell [54] demonstrated that sexting among older adolescents tended to occur in sexual and romantic contexts, whereas for younger adolescents this occurred under more platonic circumstances. Although the SHW survey question for ever having a sexually explicit image shared without consent does not explicitly ask if it was done by a romantic partner, it can be argued that if it was, then this would be classed as DRV [43], while if it was done by someone else (e.g., a friend) then it could be classed as a form of cyberbullying. As such, the crossover between cyberbullying, DRV and sexting [43,44,45,48,50,56] may help explain the significant associations found between all types of DRV, cyberbullying and sexting. Future qualitative studies are needed to explore how these risk behaviours interrelate.

### 4.1. Limitations

Due to the cross-sectional design of this study, we are unable to determine the temporal nature of the risk behaviours in relation to DRV. Nevertheless, future longitudinal studies should also explore the context in which risk behaviours interrelate with DRV, as some risk behaviours are both precursors and consequences of DRV; for example, alcohol intoxication can both make a person more vulnerable to DRV victimisation or more open to aggression and thus DRV perpetration, while drinking alcohol can also be used as a coping mechanism in response to experiencing DRV [26,32,33,34]. The wording of the SHW DRV questions do not discriminate between DRV and hurting someone in self-defence, which could potentially overestimate the prevalence of DRV perpetration or lead to DRV victims being labelled as perpetrators. Moreover, emotional DRV was described only as ‘hurtful comments’, unlike physical DRV which included several definitions. Quantifying DRV occurrence may require more nuanced measures. The pattern between reported cyberbullying victimisation and DRV victimisation and perpetration was not as clear as for reported bullying. However, we could not explore cyberbullying perpetration alongside cyberbullying victimisation. While a similar pattern to bullying perpetration is anticipated [48], more detailed measures are needed among this age group. Likewise, measures are also needed which capture longer term bullying and cyberbullying, beyond that which occurs ‘in the past couple of months’. Furthermore, as the SHW survey is only completed by young people in mainstream school, those truanting or suspended (and more likely engaging in high levels of risk behaviours) may not have completed the survey. Existing US research has highlighted higher suspension rates significantly associated with reported physical and emotional DRV victimisation [26]. In addition, self-reported data may have been biased by standard limitations (e.g., memory recall biases, social desirability, etc.). Future research should consider the inclusion of more indirect measures for the risk behaviours studied in this paper, including DRV, to reduce social desirability bias.

### 4.2. Strengths

One of the main strengths of this study is the use of a large nationally representative sample to explore multiple risk behaviours associated with DRV, findings from which have contributed to the limited number of studies on DRV in the UK. At the time of writing, there was no known research using such a large representative sample, both in the UK and internationally, that had looked at the clustering of the risk factors analysed in this paper, together with emotional and physical DRV victimisation and perpetration. This study contributes to the growing literature on DRV by demonstrating significant associations between each risk factor and DRV, while also identifying how these associations varied by gender and by emotional and physical DRV victimisation and perpetration. Nevertheless, future studies are needed that explore DRV among sexual and gender minorities as a recent review of the DRV literature suggests that lesbian, gay, bisexual, and transgender (LGBT) youth are at an increased risk for DRV [71].

## 5. Conclusions

This is one of the first studies to use a large UK nationally representative sample to identify risk behaviours associated with DRV. Our analyses revealed that students who reported bullying victimisation, bullying perpetration, cyberbullying victimisation, higher episodic drinking, ever having tried cannabis, sexting, and/or having a sexually explicit image shared without permission, had significantly higher odds of experiencing and perpetrating emotional and physical DRV. We were also able to demonstrate how the associations between certain risk behaviours differed by type of DRV (e.g., bullying perpetration and DRV perpetration) as well as how these varied by gender (e.g., higher episodic drinking and physical DRV among girls). Given that even among the youngest year groups (year 7), over half of the students who took part in the SHW survey had already dated someone, findings such as these argue for a preventative rather than reactive approach to tackling DRV. The delivery of mandatory, comprehensive and inclusive RSE lessons as part of the revised education curriculum in Wales [15,16], including a focus on healthy relationships, is a step forward. Additionally, in view of the significant associations between DRV and bullying, cyberbullying, alcohol, cannabis, and sexting, a likely impact of school-based interventions focused more holistically on supporting positive relationships and healthy school environments could be a reduction of future DRV rates as well as the other interrelated risk factors [26,62]. Nevertheless, future studies using mixed- methods are needed to explore the context in which these risk behaviours and DRV occur, to better inform such interventions.

## Figures and Tables

**Table 1:** Socio-demographic characteristics for students who have ever dated someone by gender and overall (*n*/%).

Grade (*n* = 48,397)	Male	Female	Total
**Year 7**	4515	3947	8462
	18.9%	16.1%	17.5%
**Year 8**	4868	4881	9749
	20.4%	19.9%	20.1%
**Year 9**	5126	5430	10,556
	21.4%	22.2%	21.8%
**Year 10**	4958	5247	10,205
	20.7%	21.4%	21.1%
**Year 11**	4447	4978	9425
	18.6%	20.3%	19.5%
**Family Affluence Scale (*n* = 46,402)**			
**Low**	7266	8104	15,370
	31.9%	34.3%	33.1%
**Medium**	7049	7299	14,348
	30.9%	30.9%	30.9%
**High**	8477	8207	16,684
	37.2%	34.8%	36.0%
**Ethnicity (*n* = 47,533)**			
**White**	21,349	22,495	43,844
	91.0%	93.4%	92.2%
**Black, Asian, and minority ethnic people**	2100	1589	3689
	9.0%	6.6%	7.8%
**Family structure (*n* = 45,675)**			
**Both Parents**	13,631	13,558	27,189
	61.4%	57.8%	59.5%
**Single Parent**	4190	5009	9199
	18.9%	21.4%	20.1%
**Parent & Step-Parent**	3199	3728	6927
	14.4%	15.9%	15.2%
**Other**	1195	1165	2360
	5.4%	5.0%	5.2%

**Table 2:** Prevalence rates for emotional and physical dating and relationship violence (DRV) victimisation and perpetration by gender and overall (%/n).

DRV Type	Male	Female	Total
**Emotional Victimisation**	23.6%	30.0%	26.9%
	(5384/22,839)	(7111/23,685)	(12,495/46,524)
**Physical Victimisation**	19.3%	12.8%	16.0%
	(4419/22,881)	(3039/23,678)	(7458/46,559)
**Emotional Perpetration**	18.1%	19.3%	18.7%
	(4134/22,832)	(4590/23,728)	(8724/46,560)
**Physical Perpetration**	8.1%	8.4%	8.3%
	(1857/22,886)	(1998/23,749)	(3855/46,635)

**Table 3:** Adjusted odds ratios (95% confidence intervals) for the associations between DRV and bullying, cyberbullying, sexting, alcohol, and cannabis ^1^.

Risk Behaviours	Emotional Victimisation		Physical Victimisation		Emotional Perpetration		Physical Perpetration	
	Boys (*n* = 17,856)	Girls (*n* = 18,556)	Boys (*n* = 17,867)	Girls (*n* = 18,563)	Boys (*n* = 17,855)	Girls (*n* = 18,573)	Boys (*n* = 17,866)	Girls (*n* = 18,588)
**Bullying Victimisation**								
Not been bullied	1.00 (ref)	1.00 (ref)	1.00 (ref)	1.00 (ref)	1.00 (ref)	1.00 (ref)	1.00 (ref)	1.00 (ref)
Experienced bullying	1.71 (1.55–1.89) ***	1.86 (1.73–2.01) ***	1.55 (1.41–1.72) ***	1.86 (1.64–2.11) ***	1.24 (1.11–1.38) ***	1.27 (1.16–1.39) ***	1.28 (1.10–1.48) **	1.30 (1.13–1.50) ***
**Bullying Perpetration**								
Not bullied another person	1.00 (ref)	1.00 (ref)	1.00 (ref)	1.00 (ref)	1.00 (ref)	1.00 (ref)	1.00 (ref)	1.00 (ref)
Bullied another person	1.44 (1.32–1.58) ***	1.26 (1.12–1.41) ***	1.63 (1.47–1.81) ***	1.44 (1.28–1.63) ***	2.01 (1.82–2.21) ***	1.89 (1.70–2.10) ***	2.27 (1.99–2.58) ***	1.83 (1.58–2.12) ***
**Cyberbullying Victimisation**								
Not been cyberbullied	1.00 (ref)	1.00 (ref)	1.00 (ref)	1.00 (ref)	1.00 (ref)	1.00 (ref)	1.00 (ref)	1.00 (ref)
Experienced cyberbullying	1.95 (1.77–2.15) ***	1.98 (1.82–2.15) ***	1.78 (1.59–2.00) ***	1.72 (1.53–1.92) ***	1.62 (1.47–1.79) ***	1.63 (1.46–1.82) ***	1.83 (1.57–2.14) ***	1.32 (1.13–1.53) ***
**Sexting ^2^**								
Never sexted and had no image shared	1.00 (ref)	1.00 (ref)	1.00 (ref)	1.00 (ref)	1.00 (ref)	1.00 (ref)	1.00 (ref)	1.00 (ref)
Sexted and had no image shared	2.40 (2.06–2.79) ***	2.30 (2.02–2.63) ***	2.07 (1.74–2.45) ***	2.09 (1.74–2.51) ***	2.82 (2.44–3.25) ***	2.42 (2.14–2.74) ***	2.08 (1.64–2.65) ***	2.22 (1.79–2.74) ***
Never sexted and had image shared	1.77 (1.58–1.98) ***	1.62 (1.48–1.78) ***	1.85 (1.66–2.06) ***	1.89 (1.67–2.15) ***	1.87 (1.65–2.12) ***	1.60 (1.42–1.80) ***	1.72 (1.46–2.04) ***	2.09 (1.77–2.48) ***
Sexted and had image shared	2.79 (2.41–3.22) ***	3.10 (2.75–3.49) ***	2.92 (2.51–3.40) ***	3.41 (2.92–3.99) ***	3.17 (2.72–3.69) ***	3.25 (2.85–3.71) ***	2.91 (2.40–3.53) ***	3.47 (2.82–4.27) ***
**Alcohol**								
<1 alcoholic drinks	1.00 (ref)	1.00 (ref)	1.00 (ref)	1.00 (ref)	1.00 (ref)	1.00 (ref)	1.00 (ref)	1.00 (ref)
1–4 alcoholic drinks	1.36 (1.23–1.50) ***	1.47 (1.35–1.60) ***	1.44 (1.31–1.59) ***	1.47 (1.31–1.66) ***	1.27 (1.14–1.42) ***	1.55 (1.41–1.71) ***	1.19 (1.01–1.39) *	1.66 (1.41–1.95) ***
5+ alcoholic drinks	1.49 (1.28–1.74) ***	1.73 (1.52–1.97) ***	1.75 (1.49–2.05) ***	2.04 (1.73–2.41) ***	1.36 (1.17–1.59) ***	1.84 (1.60–2.11) ***	1.35 (1.10–1.65) **	2.37 (1.94–2.89) ***
**Cannabis**								
Never tried cannabis	1.00 (ref)	1.00 (ref)	1.00 (ref)	1.00 (ref)	1.00 (ref)	1.00 (ref)	1.00 (ref)	1.00 (ref)
Tried cannabis	1.41 (1.24–1.60) ***	1.58 (1.39–1.80) ***	1.22 (1.07–1.39) **	1.61 (1.40–1.84) ***	1.60 (1.40–1.82) ***	1.66 (1.45–1.90) ***	1.20 (1.00–1.44) *	1.63 (1.38–1.94) ***

^1^ Multi-behaviour models controlled for socio-demographics which have previously been associated with DRV [9], including year group, FAS, ethnicity, and family structure. ^2^ Sexting includes measures for ‘ever having sexted’ and ‘ever having a sexually explicit image shared without consent’. * *p* < 0.05; ** *p* < 0.01; *** *p* < 0.001.

## Data Availability

The data presented in this study are available on request from the School Health Research Network (SHRN) Team in the Centre for Development, Evaluation, Complexity and Implementation in Public Health Improvement (DECIPHer) at Cardiff University. Data access requests can be made by contacting shrn@cardiff.ac.uk. Some variables are restricted to preserve the anonymity of study participants.

## Supplementary Materials

The following are available online at https://www.mdpi.com/1660-4601/18/3/1192/s1, Table S1: Unadjusted odds ratios (95% confidence intervals) for the associations between DRV and bullying, cyberbullying, sexting, alcohol, and cannabis; and Table S2: Adjusted odds ratios (95% confidence intervals) for the associations between DRV and bullying, cyberbullying, sexting, alcohol, and cannabis.
