# Supplementary material for: Risk Behaviours Associated with Dating and Relationship Violence among 11–16 Year Olds in Wales: Results from the 2019 Student Health and Wellbeing Survey
Source: Int J Environ Res Public Health. 2021 Jan 29;18(3):1192. doi: 10.3390/ijerph18031192 (PMC7908341; doi:10.3390/ijerph18031192)
Supplement: Supplementary file 1 [file ijerph-18-01192-s001.pdf]

1. **Single-Behaviour Logistic Regression Models**

Table 1. Unadjusted odds ratios (95% confidence intervals) for the associations between DRV and bullying, cyberbullying, sexting, alcohol and cannabis.

**Bullying Victimisation**			
	**Boys**	**Girls**	**All**
**Emotional Victimisation**	*n*=21,968	*n*=22,770	*n*=44,738
Not been bullied	1.00 (ref)	1.00 (ref)	1.00 (ref)
Experienced bullying	2.29 (2.14-2.45)***	2.29 (2.14-2.44)***	2.34 (2.23-2.45)***
**Physical Victimisation**	*n*=21,998	*n*=22,763	*n*=44,761
Not been bullied	1.00 (ref)	1.00 (ref)	1.00 (ref)
Experienced bullying	2.26 (2.10-2.44)***	2.64 (2.43-2.87)***	2.26 (2.14-2.40)***
**Emotional Perpetration**	n=21,965	n=22,802	n=44,767
Not been bullied	1.00 (ref)	1.00 (ref)	1.00 (ref)
Experienced bullying	1.81 (1.68-1.95)***	1.67 (1.55-1.80)***	1.74 (1.65-1.83)***
**Physical Perpetration**	*n*=22,003	*n*=22,825	*n*=44,828
Not been bullied	1.00 (ref)	1.00 (ref)	1.00 (ref)
Experienced bullying	2.28 (2.06-2.52)***	1.85 (1.69-2.03)***	2.04 (1.90-2.19)***
**Bullying Perpetration**			
	**Boys**	**Girls**	**All**
**Emotional Victimisation**	*n*=21,595	*n*=22,605	*n*=44,200
Not bullied another person	1.00 (ref)	1.00 (ref)	1.00 (ref)
Bullied another person	2.24 (2.08-2.42)***	2.03 (1.87-2.20)***	2.03 (1.93-2.14)***
**Physical Victimisation**	*n*=21,635	*n*=22,610	*n*=44,245
Not bullied another person	1.00 (ref)	1.00 (ref)	1.00 (ref)
Bullied another person	2.50 (2.31-2.70)***	2.66 (2.41-2.93)***	2.67 (2.51-2.84)***
**Emotional Perpetration**	*n*=21,595	*n*=22,649	*n*=44,244
Not bullied another person	1.00 (ref)	1.00 (ref)	1.00 (ref)
Bullied another person	2.78 (2.58-2.99)***	2.58 (2.37-2.80)***	2.63 (2.50-2.77)***
**Physical Perpetration**	*n*=21,634	*n*=22,671	*n*=44,305
Not bullied another person	1.00 (ref)	1.00 (ref)	1.00 (ref)
Bullied another person	3.37 (3.06-3.70)***	3.15 (2.81-2.54)***	3.21 (3.00-3.43)***
**Cyberbullying Victimisation**			

	Boys	Girls	All
**Emotional Victimisation**	*n*=21,891	*n*=22,742	*n*=44,633
Not been cyberbullied	1.00 (ref)	1.00 (ref)	1.00 (ref)
Experienced cyberbullying	2.97 (2.77-3.18)***	2.93 (2.76-3.12)***	3.03 (2.89-3.18)***
**Physical Victimisation**	*n*=21,929	*n*=22,736	*n*=44,665
Not been cyberbullied	1.00 (ref)	1.00 (ref)	1.00 (ref)
Experienced cyberbullying	2.86 (2.64-3.09)***	2.92 (2.72-3.13)***	2.58 (2.44-2.74)***
**Emotional Perpetration**	*n*=21,891	*n*=22,777	*n*=44,668
Not been cyberbullied	1.00 (ref)	1.00 (ref)	1.00 (ref)
Experienced cyberbullying	2.43 (2.24-2.64)***	2.23 (2.07-2.41)***	2.31 (2.18-2.44)***
**Physical Perpetration**	*n*=21,930	*n*=22,797	*n*=44,727
Not been cyberbullied	1.00 (ref)	1.00 (ref)	1.00 (ref)
Experienced cyberbullying	3.09 (2.77-3.44)***	2.10 (1.91-2.31)***	2.47 (2.29-2.67)***
		**Sexting[1]**	
	**Boys**	**Girls**	**All**
**Emotional Victimisation**	*n*=21,582	*n*=22,012	*n*=43,594
Never sexted and had no image shared	1.00 (ref)	1.00 (ref)	1.00 (ref)
Sexted and had no image shared	3.49 (3.09-3.94)***	3.98 (3.54-4.47)***	3.76 (3.45-4.09)***
Never sexted and had image shared	2.18 (1.98-2.40)***	2.21 (2.04-2.39)***	2.22 (2.08-2.36)***
Sexted and had image shared	4.45 (3.96-4.99)***	6.36 (5.76-7.02)***	5.38 (4.98-5.81)***
**Physical Victimisation**	*n*=21,605	*n*=22,010	*n*=43,615
Never sexted and had no image shared	1.00 (ref)	1.00 (ref)	1.00 (ref)
Sexted and had no image shared	2.60 (2.28-2.97)***	3.07 (2.64-3.57)***	2.70 (2.45-2.97)***
Never sexted and had image shared	2.14 (1.94-2.35)***	2.37 (2.13-2.62)***	2.13 (1.99-2.28)***
Sexted and had image shared	4.31 (3.82-4.87)***	6.28 (5.68-6.95)***	4.93 (4.52-5.38)***
**Emotional Perpetration**	*n*=21,572	*n*=22,039	*n*=43,611
Never sexted and had no image shared	1.00 (ref)	1.00 (ref)	1.00 (ref)
Sexted and had no image shared	4.27 (3.79-4.81)***	4.65 (4.10-5.27)***	4.46 (4.10-4.85)***
Never sexted and had image shared	2.40 (2.16-2.68)***	2.17 (1.96-2.41)***	2.27 (2.10-2.45)***
Sexted and had image shared	5.55 (4.93-6.25)***	7.33 (6.63-8.09)***	6.43 (5.92-6.99)***
**Physical Perpetration**	*n*=21,606	*n*=22,056	*n*=43,662

Never sexted and had no image shared	1.00 (ref)	1.00 (ref)	1.00 (ref)
Sexted and had no image shared	2.45 (2.03-2.97)***	3.33 (2.78-4.00)***	2.87 (2.51-3.28)***
Never sexted and had image shared	1.99 (1.72-2.30)***	2.56 (2.22-2.95)***	2.26 (2.05-2.48)***
Sexted and had image shared	4.57 (3.98-5.23)***	6.85 (5.99-7.85)***	5.63 (5.10-6.21)***
**Alcohol**			
	**Boys**	**Girls**	**All**
**Emotional Victimisation**	*n*=22,216	*n*=22,744	*n*=44,960
<1 alcoholic drinks	1.00 (ref)	1.00 (ref)	1.00 (ref)
1-4 alcoholic drinks	1.76 (1.63-1.91)***	2.24 (2.11-2.38)***	2.02 (1.92-2.13)***
5+ alcoholic drinks	2.84 (2.56-3.15)***	3.94 (3.60-4.30)***	3.45 (3.23-3.70)***
**Physical Victimisation**	*n*=22,254	*n*=22,738	*n*=44,992
<1 alcoholic drinks	1.00 (ref)	1.00 (ref)	1.00 (ref)
1-4 alcoholic drinks	1.47 (1.36-1.59)***	1.75 (1.61-1.89)***	1.54 (1.45-1.64)***
5+ alcoholic drinks	2.39 (2.13-2.68)***	3.35 (3.03-3.72)***	2.67 (2.44-2.93)***
**Emotional Perpetration**	*n*=22,207	*n*=22,782	*n*=44,989
<1 alcoholic drinks	1.00 (ref)	1.00 (ref)	1.00 (ref)
1-4 alcoholic drinks	1.87 (1.71-2.04)***	2.59 (2.41-2.79)***	2.21 (2.08-2.35)***
5+ alcoholic drinks	3.21 (2.89-3.56)***	4.90 (4.47-5.36)***	4.02 (3.75-4.31)***
**Physical Perpetration**	*n*=22,253	*n*=22,801	*n*=45,054
<1 alcoholic drinks	1.00 (ref)	1.00 (ref)	1.00 (ref)
1-4 alcoholic drinks	1.36 (1.21-1.52)***	1.99 (1.78-2.23)***	1.64 (1.51-1.77)***
5+ alcoholic drinks	2.29 (2.00-2.62)***	4.13 (3.69-4.63)***	3.13 (2.85-3.44)***
**Cannabis**			
	**Boys**	**Girls**	**All**
**Emotional Victimisation**	*n*=22,402	*n*=23,240	*n*=45,642
Never tried cannabis	1.00 (ref)	1.00 (ref)	1.00 (ref)
Tried cannabis	2.86 (2.63-3.11)***	3.72 (3.40-4.06)***	3.22 (3.03-3.43)***
**Physical Victimisation**	*n*=22,441	*n*=23,245	*n*=45,686
Never tried cannabis	1.00 (ref)	1.00 (ref)	1.00 (ref)
Tried cannabis	2.35 (2.14-2.57)***	3.30 (3.02-3.61)***	2.73 (2.54-2.93)***
**Emotional Perpetration**	*n*=22,394	*n*=23,279	*n*=45,673
Never tried cannabis	1.00 (ref)	1.00 (ref)	1.00 (ref)

Tried cannabis	3.54 (3.26-3.84)***	4.56 (4.16-4.99)***	4.01 (3.75-4.29)***
**Physical Perpetration**	*n*=22,443	*n*=23,305	*n*=45,748
Never tried cannabis	1.00 (ref)	1.00 (ref)	1.00 (ref)
Tried cannabis	2.46 (2.16-2.80)***	3.86 (3.48-4.28)***	3.11 (2.87-3.38)***

[1]Sexting includes measures for 'ever having sexted' and 'ever having a sexually explicit image shared without consent'.

*$p < 0.05$; **$p < 0.01$, ***$p < 0.001$

2. Multi-Behaviour Logistic Regression Models

Table 2. Adjusted odds ratios (95% confidence intervals) for the associations between DRV and bullying, cyberbullying, sexting, alcohol and cannabis[1].

	**Emotional Victimisation** Boys and girls (*n*=36,412)	**Physical Victimisation** Boys and girls (*n*=36,430)	**Emotional Perpetration** Boys and girls (*n*=36,428)	**Physical Perpetration** Boys and girls (*n*=36,454)
**Bullying Victimisation**				
Not been bullied	1.00 (ref)	1.00 (ref)	1.00 (ref)	1.00 (ref)
Experienced bullying	1.81 (1.71-1.91)***	1.59 (1.47-1.72)***	1.25 (1.16-1.34)***	1.29 (1.17-1.43)***
**Bullying Perpetration**				
Not bullied another person	1.00 (ref)	1.00 (ref)	1.00 (ref)	1.00 (ref)
Bullied another person	1.30 (1.21-1.40)***	1.70 (1.57-1.86)***	1.94 (1.81-2.08)***	2.02 (1.83-2.23)***
**Cyberbulling Victimisation**				
Not been cyberbullied	1.00 (ref)	1.00 (ref)	1.00 (ref)	1.00 (ref)
Experienced cyberbullying	2.01 (1.90-2.13)***	1.60 (1.47-1.74)***	1.62 (1.51-1.74)***	1.54 (1.37-1.73)***
**Sexting[2]**				
Never sexted and had no image shared	1.00 (ref)	1.00 (ref)	1.00 (ref)	1.00 (ref)
Sexted and had no image shared	2.36 (2.13-2.61)***	2.02 (1.79-2.27)***	2.62 (2.39-2.88)***	2.16 (1.85-2.53)***
Never sexted and had image shared	1.70 (1.58-1.83)***	1.78 (1.63-1.94)***	1.72 (1.56-1.89)***	1.91 (1.69-2.14)***
Sexted and had image shared	2.97 (2.70-3.27)***	3.04 (2.71-3.41)***	3.25 (2.93-3.61)***	3.21 (2.77-3.72)***
**Alcohol**				
<1 alcoholic drinks	1.00 (ref)	1.00 (ref)	1.00 (ref)	1.00 (ref)
1-4 alcoholic drinks	1.43 (1.34-1.53)***	1.43 (1.32-1.55)***	1.41 (1.31-1.52)***	1.41 (1.26-1.57)***
5+ alcoholic drinks	1.67 (1.52-1.83)***	1.78 (1.59-2.00)***	1.61 (1.46-1.77)***	1.87 (1.63-2.14)***
**Cannabis**				
Never tried cannabis	1.00 (ref)	1.00 (ref)	1.00 (ref)	1.00 (ref)
Tried cannabis	1.46 (1.35-1.59)***	1.42 (1.30-1.56)***	1.62 (1.47-1.78)***	1.41 (1.24-1.60)***

[1]Multipredictor models controlled for socio-demographics which have previously been associated with DRV [9], including year group, FAS, ethnicity and family structure.

[2]Sexting includes measures for 'ever having sexted' and 'ever having a sexually explicit image shared without consent'.

*$p < 0.05$; **$p < 0.01$, ***$p < 0.001$
